# Activation of CaMKIIγ potentiates T-cell acute lymphoblastic leukemia leukemogenesis via phosphorylating FOXO3a

**DOI:** 10.18632/oncotarget.20504

**Published:** 2017-08-24

**Authors:** Xudong Jiang, Zhaoxing Wu, Xiaoya Lu, Xuzhao Zhang, Qingfeng Yu, Yichao Gan, Bowen Wu, Ying Xu, Weiwei Zheng, Lei Zhang, Fei Xu, An Ma, Xiaoxian Gan, Silvia Huang, Xiaofang Yu, Wendong Huang, Rongzhen Xu

**Affiliations:** ^1^ Department of Hematology, Key Laboratory of Cancer Prevention and Intervention, China National Ministry of Education, The Second Affiliated Hospital, College of Medicine, Zhejiang University, Hangzhou 310009, China; ^2^ Cancer Institute of Zhejiang University, Hangzhou, 310009 China; ^3^ Deptartment of Clinical Laboratory of Anhui Provincial Hospital, Anhui Medical University, Hefei 230000, China; ^4^ Zhejiang Academy of Medical Sciences, Hangzhou 310009, China; ^5^ City of Hope Eugene and Ruth Roberts Summer Student Academy, City of Hope National Medical Center, Duarte, CA 91010, USA; ^6^ Molecular Oncology Program and Department of Diabetes Complications and Metabolism, Beckman Research Institute, Duarte, CA 91010, USA; ^7^ Irell & Manella Graduate School of Biological Sciences, City of Hope National Medical Center, Duarte, CA 91010, USA

**Keywords:** T-cell acute lymphoblastic leukemia, leukemogenesis, CaMKIIγ, AKT, FOXO3a

## Abstract

Ca^2+^/calmodulin–dependent protein kinase II γ (CaMKIIγ) can regulate the proliferation and differentiation of myeloid leukemia cells and accelerate chronic myeloid leukemia blast crisis, but the role of CaMKIIγ in T-cell acute lymphoblastic leukemia (T-ALL) leukemogenesis remains poorly understood. We observed that activated (autophosphorylated) CaMKIIγ was invariably present in T-ALL cell lines and in the majority of primary T-ALL samples. Overexpression of CaMKIIγ enhanced the proliferation, colony formation, *in vivo* tumorigenesis and increased DNA damage of T-ALL leukemia cells. Furthermore, inhibition of CaMKIIγ activity with a pharmacologic inhibitor, gene knock-out, dominant-negative constructs or enhancement of CaMKIIγ activity by overexpression constructs revealed that the activated CaMKIIγ could phosphorylate FOXO3a. In Jurkat cells, the activated CaMKIIγ phosphorylated FOXO3a via directly or indirectly phosphorylating AKT, excluded FOXO3a from the nucleus and inhibited its transcriptional activity. These results indicate that the activated CaMKIIγ may play a key role in T-ALL leukemogenesis, and targeting CaMKIIγ might be a value approach in the treatment of T-ALL.

## INTRODUCTION

T-cell acute lymphoblastic leukemia (T-ALL), an aggressive hematologic malignancy of developing thymocytes, comprises 10-15% of paediatric and 20-25% of adult acute lymphoblastic leukemia cases. Clinically, it is characterized by high white blood cell counts, diffuse infiltration of the bone marrow by immature T cell lymphoblasts, enlarged mediastinal lymph nodes, pleural effusions and frequent infiltration of the central nervous system during diagnosis [1, 2]. Although the introduction of intensified chemotherapy has gradually improved the clinical outcome of T-ALL over the last decades, with long-term survival rates reaching over 75% in children and about 50% in adults [3]. However, the outcome of T-ALL patients with primary resistant or early relapse remains unsatisfied. Thus, it is very important to elucidate the molecular mechanisms that cause and drive T-ALL leukemogenesis and identify novel molecular targets in order to design more specific therapies.

High frequency of phosphatase and tensin homolog (PTEN), PI3K and AKT abnormalities were identified in T-ALL [4], and aberrant activation of PI3K- AKT signaling plays a prominent role in the pathogenesis of T-ALL [5]. Activated AKT can directly phosphorylate a series of substrates, including glycogen synthase kinase β (GSK3β) [6], BCL2 associated agonist of cell death (BAD) [7], mechanistic target of rapamycin (mTOR) [8], FOXOs [9, 10]. The FOXO family of transcription factors include FOXO1, FOXO3, FOXO4 and FOXO6. FOXOs play a very pivotal role in a variety of processes including cellular differentiation [11], cell-cycle arrest [12], cell death [13], tumor suppression [14, 15], glucose metabolism [16], detoxification of reactive oxygen species (ROS) [17] and repairment of damaged DNA [18]. The AKT-mediated phosphorylation of FOXOs promotes FOXOs excluded from the nucleus, thereby repress FOXOs transcriptional function. Perturbation of FOXOs function results in deregulated cell proliferation, accumulation of DNA damage and genome instability, leading to diseases such as cancer [15, 19].

Ca^2+^/calmodulin–dependent protein kinase II (CaMKII), which consists of four different isforms (CaMKIIα, CaMKIIβ, CaMKIIγ and CaMKIIδ), is a multifunctional serine/threonine kinase. The CaMKIIα and CaMKIIβ isoforms are expressed abundantly in neurons, while the CaMKIIγ and CaMKIIδ isoforms are more widely expressed [20]. After binding Ca^2+^/calmodulin complex, autophosphorylation on Thr287 (for the β, γ, δ isoforms) or Thr286 (for the α isoform) results in Ca^2+^/calmodulin independent activity. Among the CaMKII isoforms, CaMKIIγ plays a very important role in regulating the proliferation and differentiation of myeloid leukemia cells [21, 22], and in accelerating chronic myeloid leukemia (CML) blast crisis [23]. Targeting CaMKIIγ by berbamine can eradicate imatinib mesylate-resistant CML blast crisis cells as well as leukemic stem cells [24].

Previous studies have shown the CaMKIIγ enhances T cell memory [25] and induces T cell unresponsiveness [26], but the relationship between CaMKIIγ and T-ALL leukemogenesis remains elusive. To characterize the role of CaMKIIγ in T-ALL, we evaluated whether activated (autophosphorylated) CaMKIIγ was invariably present in T-ALL and whether activated (autophosphorylated) CaMKIIγ had a role in T-ALL leukemogenesis. We observed that activated (autophosphorylated) CaMKIIγ was abundantly present in T-ALL cell lines and in the major of primary T-ALL patient samples, and overexpression of CaMKIIγ significantly promoted the growth of T-ALL cells and induced genome instability. Mechanistically, we showed that activated (autophosphorylated) CaMKIIγ inhibited FOXO3a transcriptional function via phosphorylating FOXO3a and facilitating its nuclear exclusion. These findings highlight the importance of activated CaMKIIγ in T-ALL leukemogenesis and suggest that inhibition of CaMKIIγ activity may have a significant role in the therapy of T-ALL.

## RESULTS

### Berbamine inhibits the growth of T-ALL cells partially by targeting CaMKIIγ

**Figure 1:**
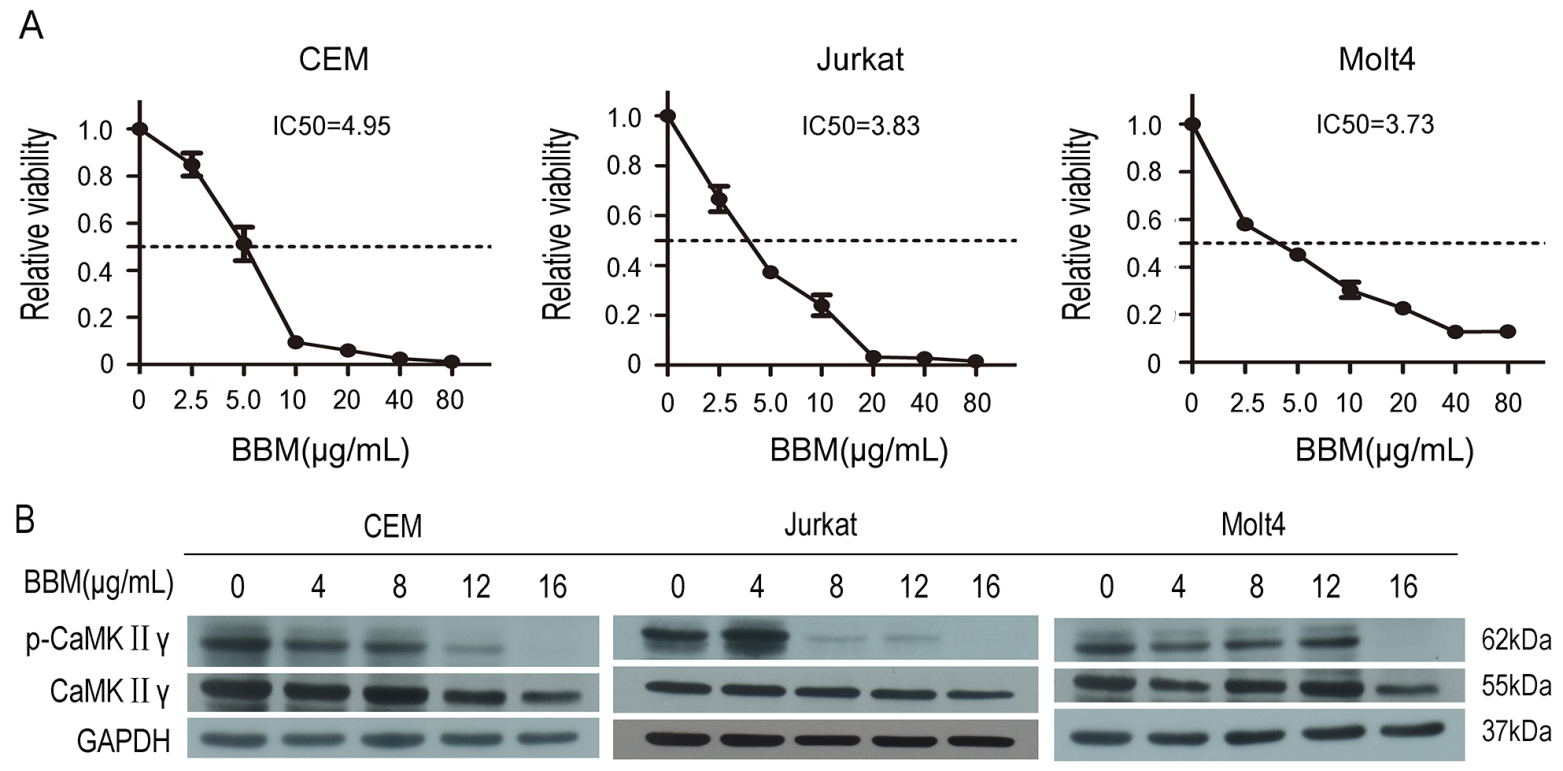
Berbamine (BBM) inhibits the growth of T-ALL cells partially by targeting CaMKIIγ **(A)** T-ALL cell lines were treated with BBM for 48 hours, and the CCK-8 assay was performed. **(B)** T-ALL cell lysates treated with BBM for 48 hours were subjected to Western blots used the CaMKIIγ, p-CaMKIIγ antibodies. GAPDH was used as a loading control.

Berbamine (BBM) is a structurally unique bisbenzylisoquinoline isolated from traditional Chinese medicine *Berberis amurensis*, and BBM exhibits significantly antiproliferative activities of myeloid leukemia [27], lymphoma [28] and multiple myeloma [29]. To determine the effect of BBM on the growth of T-ALL, Jurkat, Molt4 and CEM cell lines were treated with BBM at various concentrations for 48 hours and cell proliferation was measured. The results showed that BBM inhibited the growth of Jurkat, Molt4 and CEM cell lines, and the IC50 values were 3.83μg/mL, 3.73μg/mL and 4.95μg/mL, respectively (Figure 1A). Previous study showed that BBM targeted CaMKIIγ by blocking its ATP-binding pocket [24] and activated CaMKIIγ was a critical regulator of myeloid leukemia cell proliferation [22]. Therefore, phosphorylation of CaMKIIγ in BBM treated CEM, Jurkat and Molt4 cell lines was detected, and the results showed that phosphorylation of CaMKIIγ was inhibited in BBM treated CEM and Jurkat, but not in BBM treated Molt4 (Figure 1B). To further confirmed the effect of CaMKIIγ on the growth of T-ALL cell lines, we treated T-ALL cell lines with CaMK inhibitor KN93 and KN92 (the inactive structural analog of KN93), and examined the proliferation. KN93 showed the inhibition of all three T-ALL cell lines in a dose-dependent manner (Supplementary Figure 1, p<0.01). Taken together, these results suggest that BBM inhibits the growth of T-ALL cell lines partially by targeting CaMKIIγ.

### High expression of activated CaMKIIγ in T-ALL cells enhances proliferation, colony formation, *in vivo* tumorigenesis and increases DNA damage of leukemia cells

**Figure 2:**
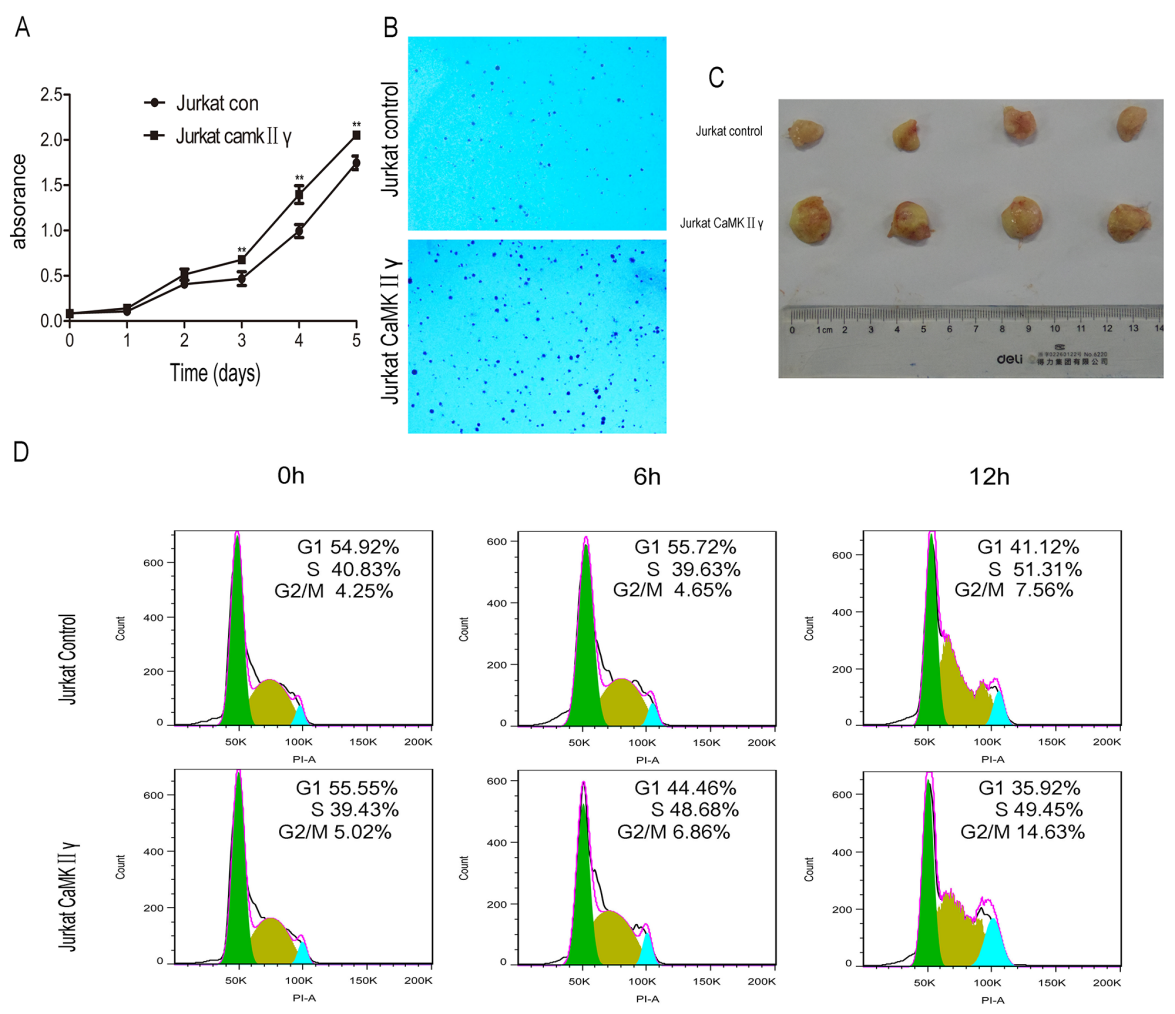
High expression of activated CaMKIIγ in T-ALL cells enhances proliferation, colony formation, *in vivo* tumorigenesis **(A)** Jurkat CaMKIIγ and Jurkat control cells were seeded in 96-well plates. The CCK-8 assays were performed at the indicated times (**p<0.01). **(B)** Jurkat CaMKIIγ and Jurkat control cells were plated in 6-well plates as described in Materials and Methods. The colonies were counted under light microscope after 2 weeks. **(C)** Representative images of xenografts. Tumor weight upon harvesting at Day 42. **(D)** Jurkat CaMKIIγ and Jurkat control cells were cultured in serum free medium for 48 hours, then stained with PI and detected by flow cytometry.

**Figure 3:**
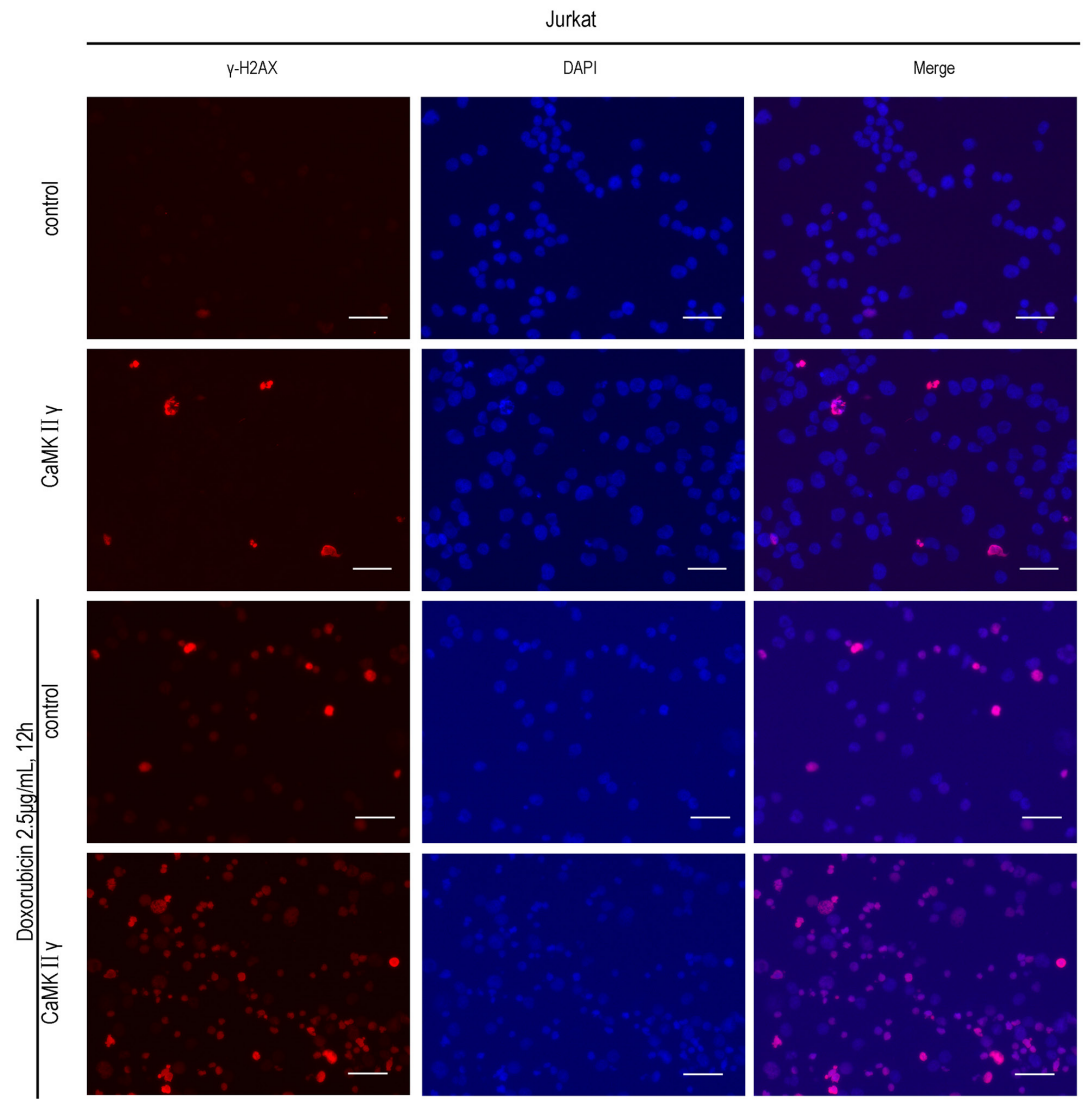
High expression of activated CaMKIIγ in T-ALL cells increases DNA damage Jurkat CaMKIIγ and Jurkat control cells were treated or untreated with doxorubicin at 2.5μg/mL for 12h, then incubated with γ-H2AX antibodies. The nuclear marker DAPI was used for nuclear location. The experiment was repeated three times, and pictures were captured from four random fields in each samples under a Zeiss Axio Vert.A1 fluorescence microscope. Scar bar represents 50μm.

The hallmarks of cancer include self-sufficiency in growth signals, enabling replicative immortality, genome instability and so on [30]. To observe the effect of activated CaMKIIγ on T-ALL leukemogenesis, Jurkat cells overexpressed full-length CaMKIIγ (Jurkat CaMKIIγ) were compared with control cells (Jurkat control), which expressed an empty vector in Jurkat, for proliferation rate, colony formation ability, *in vivo* tumorigenesis, cell cycle and DNA damage response. In contrast to control, The total and phosphorylated CaMKIIγ were higher in Jurkat CaMKIIγ (Supplementary Figure 2A). The CCK-8 assays revealed that the Jurkat cells with enforced expression of CaMKIIγ showed a higher proliferative rate compared with control cells (Figure 2A, p<0.01). To further confirmed the effect of phosphorylated CaMKIIγ on growth, we expressed dominant-negative(dn) CaMKIIγ(T287A)-FLAG in Jurkat cells. The result showed that the proliferation of Jurkat dnCaMKIIγ(T287A) cells was inhibited compared with control (Supplementary Figure 2B, p<0.01). The colony formation assays showed that cell colony significantly increased following overexpression of CaMKIIγ in Jurkat cells (Figure 2B and Supplementary Figure 2C, p<0.01). More importantly, tumor weights with CaMKIIγ overexpression in NSG (NOD/SCID/IL2Rγ-/-) mice were increased compared with control (0.78±0.05 g and 0.33±0.09 g, respectively) (Figure 2C and Supplementary Figure 2D, p<0.01). Flow cytometry analysis demonstrated that Jurkat cells with CaMKIIγ-enforced expression displayed an acceleration in cell cycle (G2/M) progression when compared with Jurkat control cells (Figure 2D). DNA double-stranded breaks can induce Histone H2AX phosphorylation on Serine 139 [31]. So we used anti phospho-Histone H2AX(Ser 139) (γH2AX) antibody to detect DNA damage. Immunofluorescence assays indicated that Jurkat CaMKIIγ cells, when treated or untreated with doxorubicin at 2.5μg/mL for 12h, had a increase in DNA damage compared with Jurkat control cells (Figure 3, Supplementary Figure 3, p<0.01). Increase in DNA damage can result in genomic alterations, such as gene deletion, gene amplification, gene insertion, etc. These alterations could be sources of genomic instability and diving forces of tumorigenesis [32, 33]. These results reveal that activated CaMKIIγ in T-ALL cells enhances proliferation, colony formation, *in vivo* tumorigenesis and increases DNA damage of leukemia cells.

### Activated CaMKIIγ phosphorylates FOXO3a by directly or indirectly phosphorylating AKT

**Figure 4:**
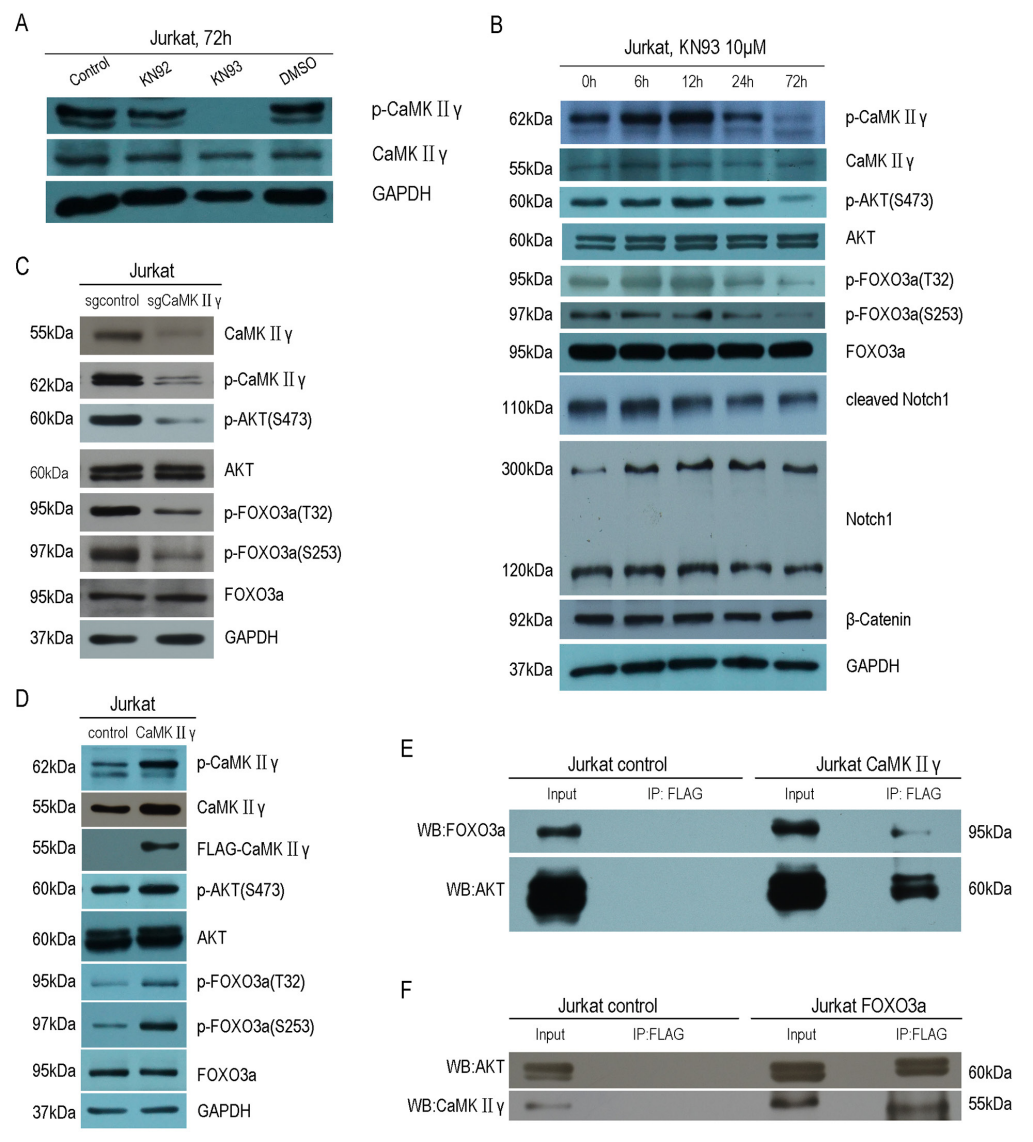
Activated CaMKIIγ phosphorylates FOXO3a by directly or indirectly phosphorylating AKT **(A)** Jurakt cells were treated with 10μM KN92, 10μM KN93, 0.1%DMSO for 72h, followed by Western blot assay for CaMKIIγ, p-CaMKIIγ antibodies. **(B)** Jurkat cells were treated with 10μM KN93 for 72h, cell lysates were subjected to Western blots with CaMKIIγ, p-CaMKIIγ, AKT, p-AKT(S473), FOXO3a, p-FOXO3a(T32), p-FOXO3a(S253), Notch1, cleaved Notch1 and β-catenin. **(C-D)** Jurkat sgCaMKIIγ and Jurkat sgcontrol, Jurkat CaMKIIγ and Jurkat control cells lysates were subjected to Western blots with CaMKIIγ, p-CaMKIIγ, AKT, p-AKT(S473), FOXO3a, p-FOXO3a(T32), p-FOXO3a(S253) antibodies. In (A-D) GAPDH was used as a loading control. **(E)** Jurkat CaMKIIγ and Jurkat control cells lysates were immunoprecipitated with FLAG antibody. The immunoprecipitates were then subjected to Western blots with AKT and FOXO3a antibodies. **(F)** Jurkat FOXO3a and Jurkat control cells lysates were immunoprecipitated with FLAG antibody. The immunoprecipitates were then subjected to Western blots with AKT and CaMKIIγ antibodies.

To explore the molecular mechanism of activated CaMKIIγ in T-ALL leukemogenesis, we treated with the CaMK inhibitor KN93 and BBM, knocked out CaMKIIγ with CRISPR/Cas9 system, expressed full-length CaMKIIγ-FLAG and dnCaMKIIγ(T287A)-FLAG in Jurkat cells. As shown in Figure 4A, there were no difference about the levels of CaMKIIγ phosphorylation in 10μM KN92 treatment of Jurkat cells and 0.1% DMSO treatment for 72h, when compared with Jurkat cells. But the levels of CaMKIIγ phosphorylation in 10μM KN93 treated Jurkat cells were not detected, and there was a time-dependent reduction in CaMKIIγ phosphorylation (Figure 4B). This decrease was associated with a reduction in the phosphorylation of AKT(S473), FOXO3a(T32, S253), whereas the total levels of these phosphorylated proteins had not significant difference (Figure 4B). Notch signalling play a key role in the development and maintenance of T-ALL [34] and previous study indicated that CaMKIIγ up-regulated β-catenin via phosphorylating GSK3β [21]. So we detected the levels of Notch1, cleaved Notch1 and β-catenin in KN93 treatment of Jurkat cells, and their levels did not change (Figure 4B). These observations indicated that, in Jurkat cells, CaMKIIγ was involved in increasing the phosphorylation of AKT, FOXO3a. Furthermore, the phosphorylation of CaMKIIγ, AKT(S473), FOXO3a(T32, S253) were inhibited in Jurkat cells treated with BBM for 48 hours in a dose-dependent manner, and the total level of CaMKIIγ, AKT, FOXO3a did not change (Figure 1B and Supplementary Figure 4A). In CRISPR/Cas9 system, the phosphorylation of AKT(S473), FOXO3a(T32, S253) were declined following CaMKIIγ reduction, and the level of total proteins did not change (Figure 4C). Same results could be observed in Jurkat cells expressed with dominant-negative CaMKIIγ constructs (Jurkat dnCaMKIIγ(T287A)) (Supplementary Figure 4B). In addition, the phosphorylation of AKT(S473), FOXO3a(T32, S253) were higher in Jurkat CaMKIIγ cells, when compared with control, and the level of total proteins did not change (Figure 4D)

To obtain the biochemical evidence for an interaction among CaMKIIγ, AKT and FOXO3a, we performed co-immunoprecipitation assays. We found that CaMKIIγ, AKT and FOXO3a protein were present in a protein complex immunoprecipitated by the Flag antibody in Jurkat CaMKIIγ cells and Jurkat control cells (Figure 4E). For reciprocal immunoprecipitate, we transduced full-length FOXO3a-FLAG in Jurkat cells (Jurkat FOXO3a). As we expected, CaMKIIγ, AKT and FOXO3a protein were also present in a protein complex immunoprecipitated by the Flag antibody in Jurkat FOXO3a cells and Jurkat control cells (Figure 4F). To demonstrate the relationship among CaMKIIγ, AKT and FOXO3a was not only in Jurkat cells, we expressed full-length CaMKIIγ-FLAG in HEK293 cells and similar results could be observerd in HEK293 (Supplementary Figure 4C and 4D). Taken together, these data show that activated CaMKIIγ phophorylates FOXO3a by directly or indirectly phophorylating AKT.

### CaMKIIγ-mediated phosphorylation of FOXO3a excludes FOXO3a from the nucleus and reduces its transcriptional activity

**Figure 5:**
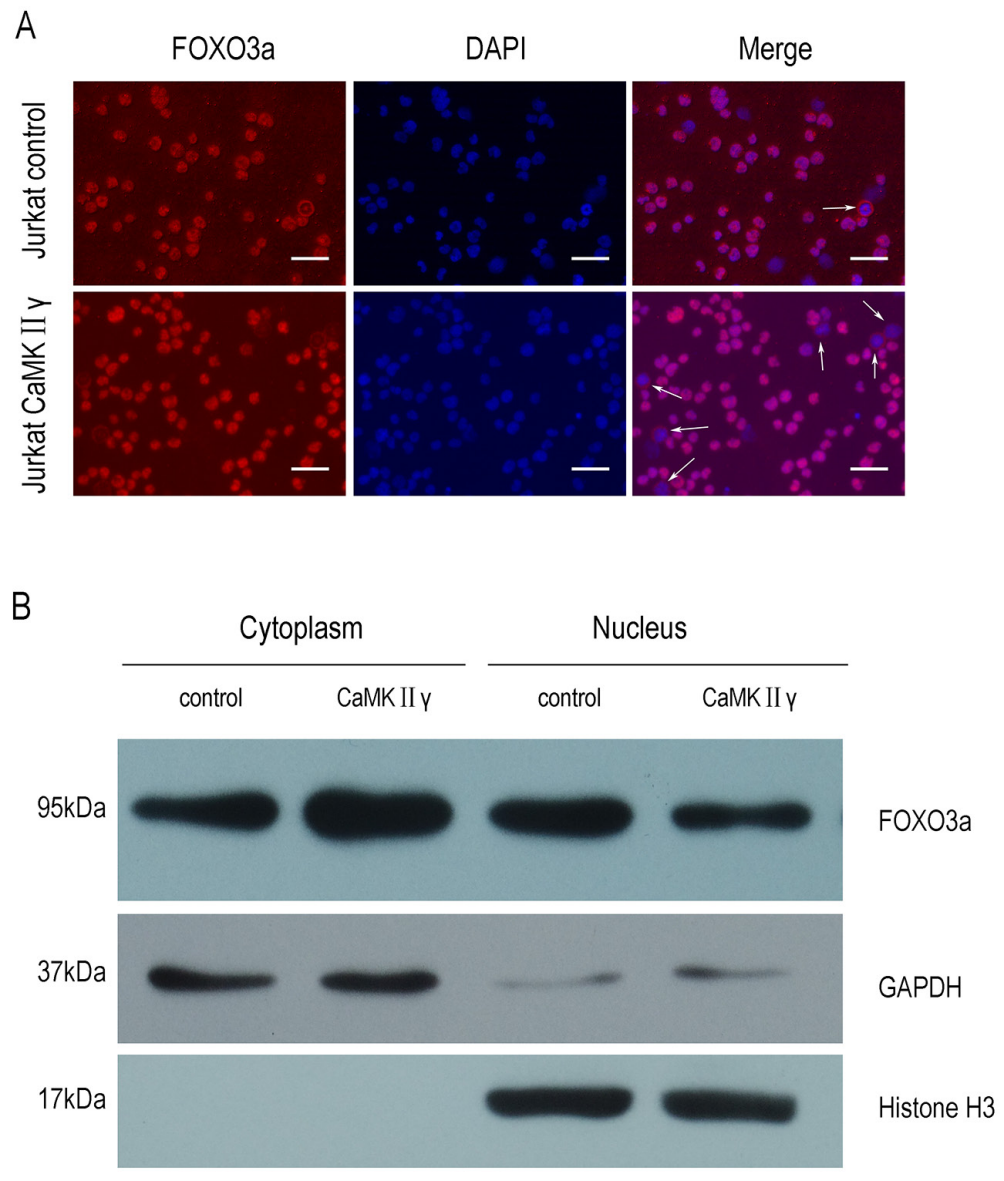
CaMKIIγ-mediated phosphorylating FOXO3a excludes it from the nucleus **(A)** Jurkat CaMKIIγ and Jurkat control cells were incubated with FOXO3a antibodies. The nuclear marker DAPI was used for nuclear location. The experiment was repeated three times, and pictures were captured from four random fields in each samples under a Zeiss Axio Vert.A1 fluorescence microscope. The arrows in the merged images indicated cells in which cytoplamic FOXO3a protein was observed. Scar bar represents 50μm. **(B)** Jurkat CaMKIIγ and Jurkat control cell fraction were done as described in Materials and Methods. The fractions were then subjected to Western blots with FOXO3a antibodies. GAPDH and Histone H3 were used as a loading control.

**Figure 6:**
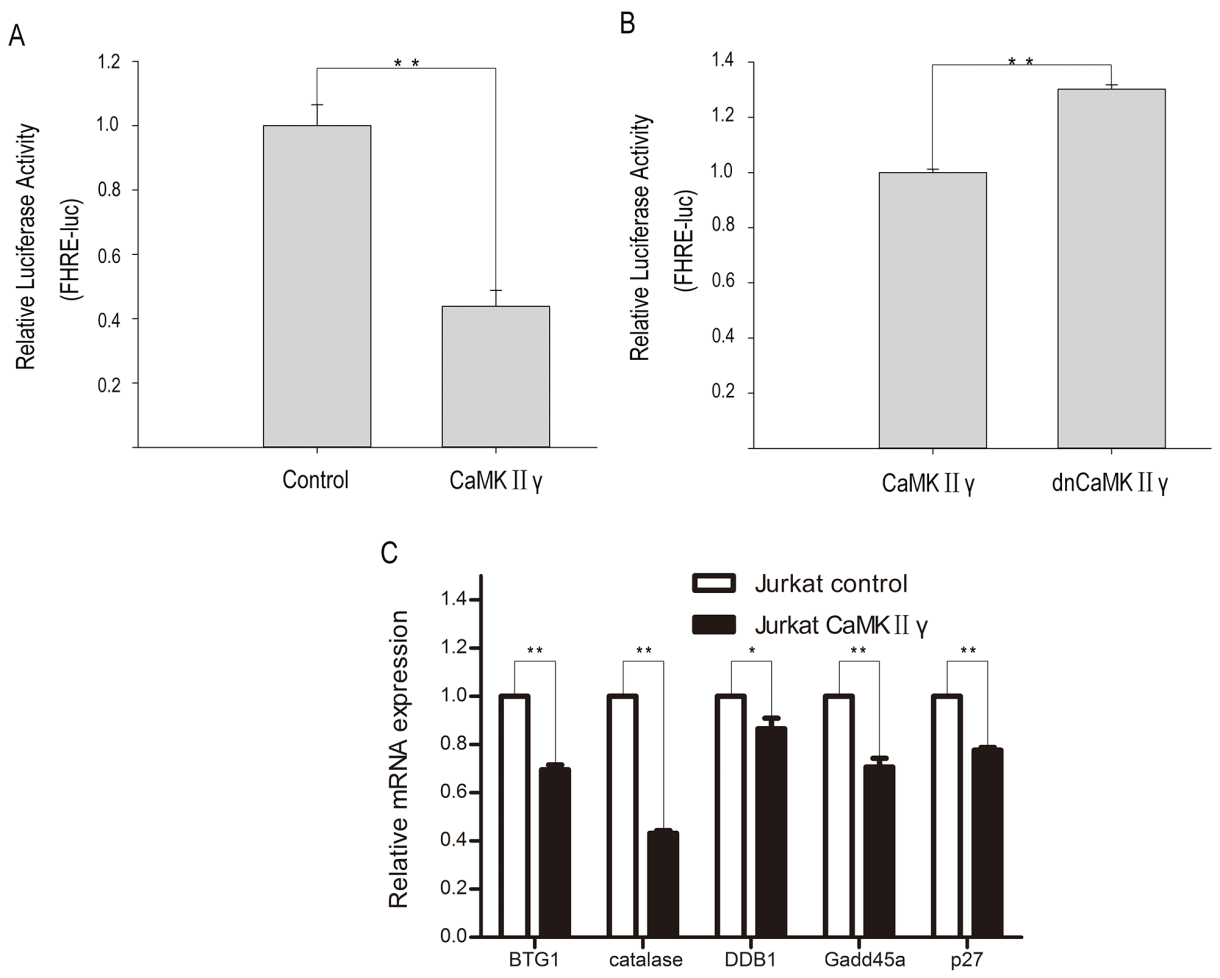
CaMKIIγ-mediated phophorylating FOXO3a reduces its transcriptional activity **(A-B)** HEK293 were cotransfection with p4xFHRE-luc, pSV-β-Galactosidase control vector and CaMKIIγ full-length expression vector or CaMKIIγ dominant-negative expression vector or control vector. The relative luciferase activity was assayed at 48 hours after transfection (**p<0.01). **(C)** qPCR analysis of BTG1, catalase, DDB1, Gadd45a and p27 were performed on RNA isolated from Jurkat CaMKIIγ and Jurkat control cells. The relative expression levels of target mRNA were normalized to those of β-actin mRNA (**p<0.01,*p<0.05).

As above mentioned, CaMKIIγ can phosphorylate FOXO3a. In order to elucidate the influence of CaMKIIγ-mediated phosphorylation of FOXO3a on its subcellular localization and its transcriptional activity, we performed the immunofluorescence assay, cell fractions assay, luciferase reporter assay and realtime PCR (qPCR). As shown in Figure 5A, there was an increase in FOXO3a protein in the cytoplasm in Jurkat CaMKIIγ cells, compared with Jurkat control cells. Similarly, cell fractions assay showed that Jurkat CaMKIIγ cells displayed a considerable increase in FOXO3a protein in the cytoplasm when compared with Jurkat control cells (Figure 5B). In luciferase reporter assay, we observed that cotransfection of the p4xFHRE(Forkhead family responsive element)-luc vector with the expression vector harboring CaMKIIγ decreased FHRE reporter activity in HEK293 cells as compared with the control vector transduced HEK293 cells (Figure 6A). Moreover, FHRE reporter activity had a significant increase in cotransfection of the p4xFHRE-luc vector with the expression vector harboring dnCaMKIIγ(T287A) in HEK293 cells, when compared with HEK293 cells co-transfecting of the p4xFHRE-luc vector with the expression vector harboring CaMKIIγ (Figure 6B). FOXO3a can transcript many genes directly, for example, BTG1 associated with cell differentiation [11], catalase involved in oxidative stress [35, 36], DDB1 associated with nucleotide-excision repair [37], Gadd45a involved in DNA damage repair [18], p27 associated with cell cycle arrest [38]. In order to further confirm the effect of CaMKIIγ on transcriptional activity of FOXO3a, we chosed these five target genes of FOXO3a and detected their mRNA levels by qPCR. As shown in Figure 6C, the levels of their mRNA decreased in Jurkat CaMKIIγ cells compared with control. DNA repair is stimulated by the FOXO3a through Gdd45a [18], so the reduction of Gdd45a can inhibit DNA repairment and increase DNA damage. Maybe this could account of what we observed in Figure 3. Taken together, these data show that CaMKIIγ-mediated phosphorylation of FOXO3a excludes FOXO3a from the nucleus and reduces its transcriptional activity.

### Activated CaMKIIγ is present in primary T-ALL cells and associated with high white blood cell count

**Figure 7:**
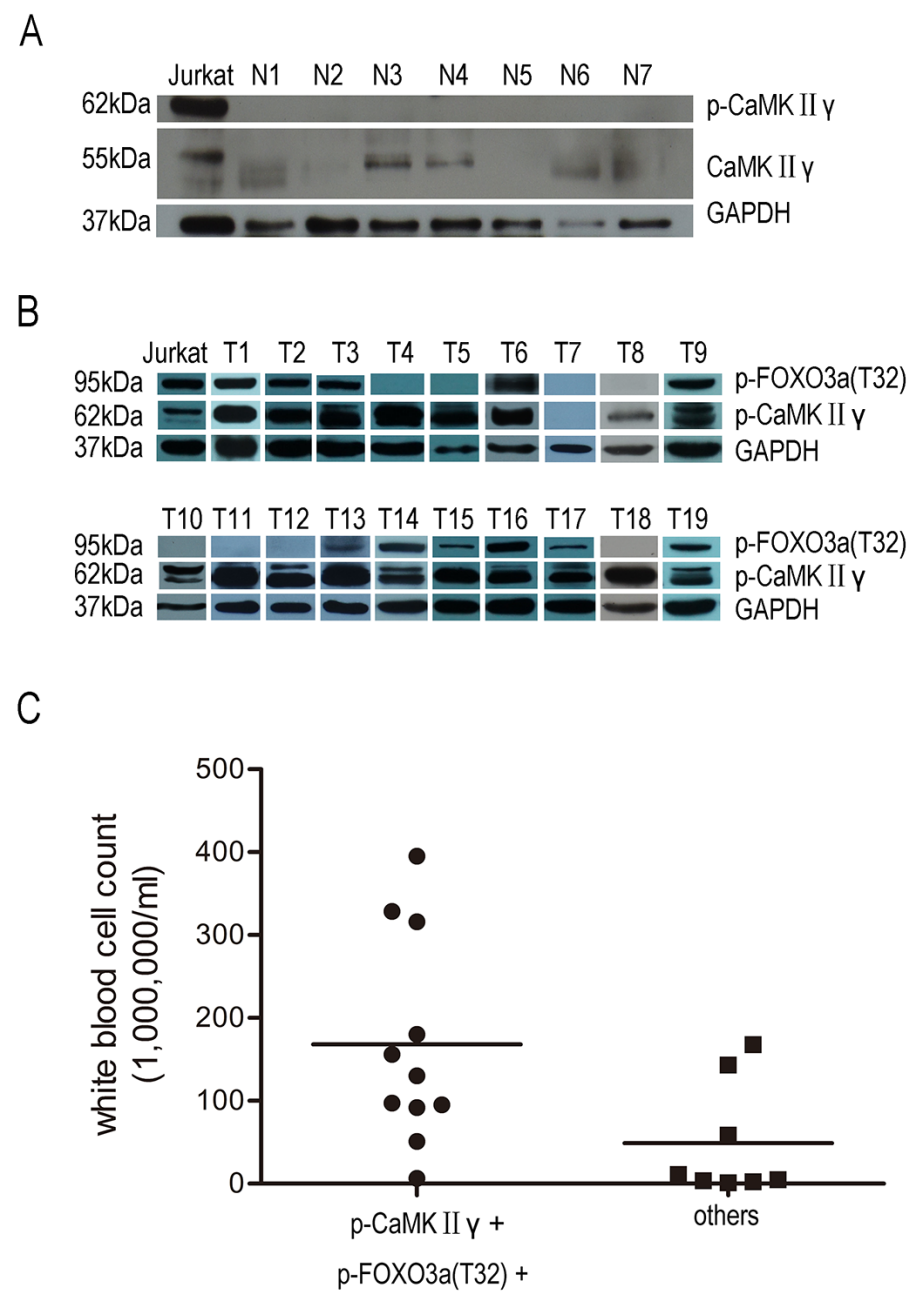
Activated CaMKIIγ is present in primary T-ALL cells not in PBMC **(A)** PBMC lysates were subjected to Western blots with CaMKIIγ, p-CaMKIIγ. **(B)** Primary T-ALL celllysates were subjected to Western blots with p-CaMKIIγ, p-FOXO3a(T32). In A-B, GAPDH was used as a loading control. **(C)** Correlation between p-CaMKIIγ, p-FOXO3a(T32) protein and white blood cell count in primary T-ALL (p<0.05).

These observations mentioned above promoted us to detect the expression of CaMKIIγ/FOXO3a in primary T-ALL cells and explore the relevance of p-CaMKIIγ/p-FOXO3a(T32) and clinical features of T-ALL. Western blot analysis showed that the total CaMKIIγ proteins were low and p-CaMKIIγ could not be observed in normal peripheral blood mononuclear cells(PBMC) (Figure 7A). In contrast to normal PBMC and Jurkat, 18/19(94.7%) exhibited high expression of p-CaMKIIγ protein in primary T-ALL (Figure 7B, Table1). The expression of p-FOXO3a(T32) protein was also performed, and the results showed 8/19(42.1%) were high, 3/19(15.8%) were weak and 8/19(42.1%) were negative respectively (Figure 7B, Table 1). Furthermore, we explored the potential associations between p-CaMKIIγ/p-FOXO3a(T32) expression and white blood cell count, bone marrow blast cells(%) (Table 1). And the results showed patients with both p-CaMKIIγ and p-FOXO3a(T32) positive showed higher white blood cell count than patients with p-CaMKIIγ negative or p-CaMKIIγ positive and p-FOXO3a(T32) negative (Figure 7C, p=0.027). Taken together, positive expression of p-CaMKIIγ and p-FOXO3a(T32) significantly correlates with higher white blood cell count.

**Table 1:** Correlation between p-CaMKIIγ, p-FOXO3a(T32) protein and white blood cell count in primary T-ALL

No.	Bone Marrow blast cells(%)	White blood cell count (10^6^/ml)	Relative expression of p-CaMKIIγ	Relative expression of p-FOXO3a(T32)
T1	93	315.9	1.21	0.57
T2	90	156	0.89	0.38
T3	89	6.4	0.87	0.46
T4	81	2.2	1.40	0
T5	89.5	143	3.55	0
T6	87.5	51.1	2.87	1.28
T7	84	10.9	0	0
T8	71	1.1	0.8	0
T9	56.5	97.45	1.49	0.59
T10	70	3.36	2.28	0
T11	70.5	58.5	1.77	0
T12	95	167.5	2.13	0
T13	96	328.5	1.74	0.07
T14	88	395	0.58	0.35
T15	86	130.2	1.34	0.09
T16	94	95.2	0.88	0.66
T17	60	91.7	0.78	0.07
T18	77	4.6	3.27	0
T19	84	180	0.46	0.39

Relative expression of p-CaMKIIγ: Grey scale of p-CaMKIIγ/Grey scale of GAPDH.

Relative expression of p-FOXO3a(T32): Grey scale of p-FOXO3a(T32)/Grey scale of GAPDH.

More than 0.2 of relative p-CaMKIIγ and relative p-FOXO3a(T32) means high.

## DISCUSSION

CaMKIIs are multifunctional serine/threonine protein kinases that respond to the fluctuation of calcium. The previous studies have revealed that CaMKIIs play important roles in many aspects. In the neural system, CaMKIIα and CaMKIIβ are necessary for long-term potentiation induction, which is thought to underlie some forms of learning and memory [39]. In the circulation system, CaMKIIδ is associated with hypertrophy, arrhythmias and myocyte apoptosis [40]. In the immune system, CaMKIIγ has influence on CD8^+^T cell proliferation, immature T cell lifespan and T cell memory [25, 26]. Our previous study and other data show that CaMKIIγ can regulate the proliferation and differentiation of AML [21, 22], promote survival and self-renewal of leukemia stem cell and accelerate blast crisis of CML [23, 24]. In this report, we define activated CaMKIIγ as an important regulator of T-ALL leukemogenesis. Indeed, we observe that activated CaMKIIγ are invariably present in T-ALL cell lines and also present in the majority of primary T-ALL patient samples. More importantly, our study reveals that CaMKIIγ can phosphorylate FOXO3a, exclude FOXO3a from the nucleus and inhibit FOXO3a transcriptional function.

What are the molecular mechanisms that trigger and sustain CaMKIIγ constitutive activation in the T-ALL cell lines and primary T-ALL samples? T-ALL cells are remarkably heterogenous and diverse molecular mechanisms may trigger CaMKIIγ activation in T-ALL. The primary trigger of CaMKIIγ activation is its binding to Ca^2+^/calmodulin, and the levels of Ca^2+^/calmodulin are regulated by intracellular Ca^2+^ concentration. In T lymphocytes, following binding of antigen/MHC complexes to the T cell receptor, intracellular channels release Ca^2+^ from intracellular stores, and by depleting the stores trigger prolonged Ca^2+^ influx through store-operated Ca^2+^ channels in the plasma membrane [41]. So sustain stimulation of exogenous and endogenous antigen can maintain CaMKIIγ constitutive activation in T cell. Furthermore, ROS, which can stimulate the development of cancer [42] and can be produced by tumor cells [43], also can increase intracellular Ca^2+^ concentration and maintain CaMKIIγ activation [44, 45]. In addition, calcium- independent activation of CaMKII has also been observered. For example, ROS can maintain CaMKII constitutively activation via oxidation of methionines 281/282 [46], and the activity of protein phosphatases 2A (PP2A), a phosphatase known to dephosphorylate CaMKII [47], is inhibited by oxidants in Jurkat cells [48]. Thus, many molecular events include endogenous and exogenous factors maintain CaMKIIγ constitutive activation in the T-ALL.

What are the key substrates of CaMKIIγ that are involved in regulating T-ALL leukemogenesis? Retinoic acid receptors(RARa), an important regulator of the terminal differentiation of acute promyelocytic leukemia cells, is directly phosphorylates by CaMKIIγ, and this phosphorylation inhibits the RARa transcriptional activity [22]. In myeloid leukemia cells, CaMKIIγ directly phosphorylates and enhances signal transducers and activators of transcription 3 (Stat3) transcriptional function [21]. Our previous study reveals that CaMKIIγ directly or indirectly phosphorylates the CDK inhibitor p27Kip1 and accelerates transition of both G_0_–G_1_ and S–G_2_/M in cell cycles in CML cells [23]. We also observe that CaMKIIγ activates nuclear factor kappa B (NF-κB), β-catenin and Stat3 networks, which are essential for survival and self-renewal of LSC in CML cells [24]. In Jurkat cells, upon hydrogen peroxide- or TCR/CD3- or phorbol ester-induced, CaMKII can phophorylate IκB kinase (IKK), a kinase that phophorylate inhibitor of kappa B (IκB), then release NF-κB, an important transcription factor involved in oncogenesis [49], translocate NF-κB to the nuclus where NF-κB acts as a transcription factor [45, 50]. Our present study indicates that CaMKIIγ can phosphorylate FOXO3a by directly or indirectly phosphorylating AKT, reduce nuclear FOXO3a and inhibit transcription of FOXO3a target gene. Furthermore, CaMKII can regulate phosphorylation of ERK1/2 and AKT, which are cancer-associated signaling pathways [51–53]. In addition, previous study show that AKT, IKK and ERK1/2 can phosphorylate FOXO3a and inhibit FOXO3a transcriptional activity [10, 54, 55]. Taken together, CaMKIIγ activity in regulating T-ALL leukemogenesis seems to orchestrate a complex interacting network of molecular events that are involved in oncogenesis.

Our observation that CaMKIIγ potentiates T-ALL leukemogenesis via phosphorylating FOXO3a indicates that targeting CaMKIIγ might be of therapeutic benefit in human T-ALL. Berbamine, isolates from traditional Chinese medicine, targets CaMKIIγ by blocking its ATP-binding pocket and exerts activity in inhibiting CML and liver cancer cell proliferation [24, 56]. Thus, Berbamine and its analogs may be of significant value in the treatment of T-ALL.

## MATERIALS AND METHODS

### Cells and cell culture

Jurkat, Molt4, CEM, HEK293 and 293T cells were purchased from the American Type Culture Collection (ATCC, Manassas, VA, USA). Jurkat, Molt4, CEM cells were grown in complete RPMI 1640 (GIBCO, Bethesda, MD, USA) supplemented with 10% fetal bovine serum (FBS), 100 μg/mL streptomycin and 100 units/mL penicillin. HEK293 and 293T cells were grown in complete DMEM (GIBCO, Bethesda, MD, USA) supplemented with 10%FBS, 100 μg/mL streptomycin, and 100 units/mL penicillin. All five cell lines were maintained at 37°C with 5%CO_2_.

### Reagents and antibodies

Phospho-CaMKII (Thr286 and Thr287 ) antibody was purchased from Santa Cruz Biotechnology (Santa Cruz, CA, USA). Phospho-AKT (Ser473), AKT (pan), Phospho-FOXO3a (Thr32), Phospho-FOXO3a (Ser253), FOXO3a, Notch1, cleaved Notch1, β-catenin, γ-H2AX, GAPDH, Histone H3 antibodies were obtained from Cell Signaling Technology (Beverly, MA, USA). CaMKIIγ antibody was purchased from ABGENT (Wuxi, China). FLAG antibody was purchased from Sigma-Aldrich (St.Louis, MO, USA). KN92 and KN93 were obtained from Calbiochem (San Diego, CA, USA). Other chemical reagents were purchased from Sigma-Aldrich (St.Louis, MO, USA) or Fisher Scientific (Pittsburgh, PA, USA) unless specified.

### Ethics statement

Normal peripheral blood mononuclear cells mononuclear cells (PBMC) and primary T-ALL cell samples were isolated from healthy volunteers or T-ALL patients with their written informed consent in accordance with the Declaration of Helsinki. All experiments were approved by the ethics committee of Second Affiliated Hospital, College of Medicine, Zhejiang University.

### Plasmid construction

The human CaMKIIγ coding sequence (NM_172171.2) or FOXO3a coding sequence (NM_001455.3) with a 3×FLAG sequence and a kozak sequence was cloned into the vector pCDH-MSCV-MCS-EF1α-GFP+Puro (System Biosciences, Palo Alto, CA, USA) using Hieff Clone™ One Step Cloning Kit (Yeasen, Shanghai, China). Point mutation of CaMKIIγ T287A was produced using Hieff Mut™ Site-Directed Mutagenesis Kit (Yeasen, Shanghai, China) with pCDH-MSCV-CaMKIIγ+3×FLAG-EF1α-GFP+Puro as template, and the primers as follows (mutated base in lower case): forward, 5’-TCGTCA GGAGgCTGTGGAGTGTTTGCGCAAGTTCAATGCCCG-3’; revese, 5’-CACT CCACAGCCTCCTGACGATGCATCATGGATGCCACCGTG-3’.

For knockout of CaMKIIγ, we used CRISPR/Cas9 system. Two seperate sgRNAs against CaMKIIγ were designed and cloned into lentiCRISPR V2 vector (Addgene plasmid # 52961, Cambridge, MA, USA) following the the CRISPR protocol [57]. Then we evaluated them in HEK293 cells for their ability to knockdown CaMKIIγ. The sequence of the more efficacious sgRNA to target CaMKIIγ as follows: 5’-CACCGTGCTTTCTCTGTGGTCCGC-3’; 5’-AAACGC GGACCACAGAGAAAGCAC-3’.

### Lentiviral infection

Lentiviral infections were carried out according to the standard procedures. In brief, 293T cells were co-transfected with viral packaging vectors psPAX2 and pMD2.G (Addgene, Cambridge, MA, USA), along with a lentiviral construct expressing vector or the empty vector as control, using Polyjet transfection reagent (SignaGen, Rockville, MD, USA) according to the manufacturer’s protocol. The transfection medium was replaced after 6 hours with fresh DMEM, and 48 hours later the viral supernatants were collected. The viral supernatant was added along with 1μg/mL Polybrene, and cells were incubated at 37°C in 5% CO_2_. The medium was replaced after 24 hours with fresh RPMI 1640 supplement with 10%FBS, and 72 hours later 2μg/mL puromycin was added to the infected cells for selection.

### Cell survival/proliferation assay

Cells were seeded in 96-well microtitre plates and survival/ proliferation was measured using the Cell Counting Kit-8 (CCK-8) from Dojindo (Kumamoto, Japan).

### Colony formation assay

Cells (5000 cells per plate) were suspended in 1.5 mL of 2×RPMI 1640 with 20%FBS, and mixed with 1.5 mL of 0.7% low-melting- point agarose in PBS. Then cells were plated on a bottom layer containing 0.5% agarose and 10% FBS in 6-well plate. After 2 weeks, the colonies were counted under light microscope.

### Xenograft

All animal procedures were approved by the institution’s ethics committee.

To establish xenograft model, female NSG (NOD/SCID/IL2Rγ-/-) mice (n=4 per group, 5-weeks) were injected subcutaneously in the left flank with 10^7^ Jurkat CaMKIIγ cells or Jurkat control cells in suspension. Tumor weights were measured at the end of experiments (6-weeks).

### Cell cycle assay

Cells were harvested and fixed with 75% ethanol overnight at 4°C, incubated with propidium iodide (PI) staining solution for 30 min, and then detected by flow cytometry.

### Immunofluorescence assay

Cells were harvested and fixed with 4% paraformaldehyde for almost 30 min, then permeabilized with 0.1% Triton X-100 for 10 min and blocked with PBS containing 5% goat serum for 30 min at room temperature. Staining of cells with antibody was performed overnight at 4°C in a humidity box. Then cells were incubated with a Rodamine-conjugated polyclonal antibody for 1 h at room temperature. After three washes with PBST (pH 7.2), the slides were counterstained with 4′,6′-diamidino-2-phenylindole (DAPI) and sealed. Fluorescence was observed with a Zeiss Axio Vert.A1.

### Cell fractionation

Cells were harvested with Buffer A ( 10 mM Hepes, pH 7.9, 2.5 mM MgCl_2_, 10 mM KCI,1mM dithiothreitol, protease and phosphatase inhibitors), incubated on ice for 15 min and centrifuged with 8,000g for 5 min at 4°C to obtain the supernatant as the cytoplasmic fraction. The precipitates were washed with Buffer A three times, resuspended in Buffer B (20 mM HEPES, pH 7.9, 25% (vol/vol) glycerol, 420 mM NaCl, 2.5 mM MgCl_2_, 0.2 mM EDTA,1mM dithiothreitol, protease and phosphatase inhibitors) and incubated on ice for 20 min. Centrifugation was performed and the supernatant was obtained as the nuclear fraction.

### Co-immunoprecipitation

For co-immunoprecipitation, cell extracts composed of 5.0×10^7^ cells were prepared by solubilization in 1ml cell lysis buffer (0.5% NP-40, 10 mM NaCl, 10 mM Tris-HCl, pH 8.0, 0.3M Sucrose, 3 mM MgCl_2_, phosphatase and protease inhibitors cocktail) for 15 min at 4°C. Centrifuge the lysate at 10,000 g for 10 min at 4°C, and the cell extract was immunoprecipitated with 7.5 μg antibodies against Flag and incubated with 50 μL of protein A Magnetic Beads (Bio-Rad, Hercules, CA, USA) overnight at 4 °C by continuous inversion. Immunocomplexes were pelleted and washed three times with PBST. The precipitated immunocomplexes were boiled in 2×Laemmli buffer.

### Western blot analysis

Cell lysates were subjected to sodium dodecyl sulfate– polyacrylamide (SDS–PAGE) gels (Bio-Rad), electrophoresed and then transferred to polyvinylidene fluoride (PVDF, Bio-Rad) membranes and blocked with 5% nonfat milk in TBS–Tween 20 (TBS-T) and then reacted with primary antibodies overnight at 4°C. After washing three times with TBS-T, membranes were reacted with a horseradish peroxidase–conjugated secondary antibody for 1 h at room temperature and developed with chemiluminescence.

### RNA extraction and real-time PCR

Total RNA was purified from cells using Trizol reagent (Invitrogen, Carlsbad, CA, USA) according to the manufacturer’s instructions. 500 ng of total RNA was reverse transcribed to cDNA using the PrimeScript^™^ RT reagent Kit (Perfect Real Time) (Takara, Tokyo, Japan). For Real-Time PCR (qPCR), primers used were as follows: BTG1 forwad, 5’-AACGAGCCCTTCCAAAAACT-3’; BTG1 reverse, 5’-TCCATAATCCATCCCCA AGA-3’; catalase forward, 5’-TTCGGTTCTCCACTGTTGC-3’; catalase reverse, 5’-AATGGGGGTGTTATT TCCAA-3’; DDB1 forward, 5’-GCGGGCTTCATAGAGACTTG-3’; DDB1 reverse, 5’-ACAACTGGCAACACCAATCA-3’; Gadd45a forward, 5’-GGCCC GGAGATAGATGACTT-3’; Gadd45a reverse, 5’-TTTTCCTTCCTGCATGGTTC-3’; p27 forward, 5’-GCCCTCCCCAGTCTCTCTTA-3’; p27 reverse, 5’-TCAAA ACTCCCAAGCACCTC-3’. The relative expression levels of target mRNA were normalized to those of β-actin mRNA. qPCR analyses were employed with SYBR® Premix Ex Taq™ II (Tli RNaseH Plus) (Takara) on Stepone plus™ Real-Time PCR Systems (Applied Biosystems, Foster City, CA, USA).

### The luciferase reporter assay

The reporters described here were derived from the pGL2-promoter luciferase vector (Promega, Madison, WI, USA). Reporters containing the Forkhead family responsive element (FHRE) were generated by ligation of concatemerized oligonucleotides to *Kpni-Xhoi*- digested pGL2-promoter. They were designated p4xFHRE-luc (with four copies of FHRE). The complementary oligonucleotides used for pFHRE-luc constructions were 5’-CA AGTAAACAACTATGTAAACAACTATGTAAACAACTATGTAAACAAC-3’ and 5’-TCGAGTTGTTTACATAGTTGTTTACATAGTTGTTTACATAGTTGTTTACTTGGTAC-3’. HEK293 cells grown in 24-well dishes were cotransfected with 200ng of p4x FHRE-luc vector, 20 ng of pSV-β-Galactosidase control vector**,** as an internal control, and 200 ng of CaMKIIγ full-length expression vector or CaMKIIγ dominant-negative expression vector or control vector. The relative luciferase activity was assayed according to the Promega protocol at 48 hours after transfection.

### Statistical analysis

Results are shown as means ± SD. Differences were evaluated by t-test analysis of variance and P-values <0.05 were considered statistically significant.

## SUPPLEMENTARY MATERIALS FIGURES


