# Supplementary material for: Activation of CaMKIIγ potentiates T-cell acute lymphoblastic leukemia leukemogenesis via phosphorylating FOXO3a
Source: Oncotarget. 2017 Aug 24;8(43):75050–64. doi: 10.18632/oncotarget.20504 (PMC5650399; doi:10.18632/oncotarget.20504)
Supplement: Supplementary file 1 [file oncotarget-08-75050-s001.pdf]

# Activation of CaMKIIγ potentiates T-cell acute lymphoblastic leukemia leukemogenesis via phosphorylating FOXO3a

## SUPPLEMENTARY MATERIALS

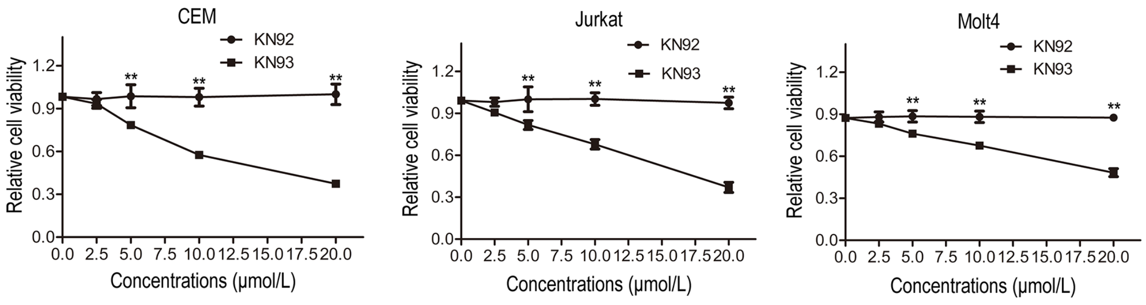


**Supplementary Figure 1: CEM, Jurkat, Molt4 were treated with various concentrations of KN93, KN92 for 72h, and the CCK-8 assay was performed (**p<0.01).**

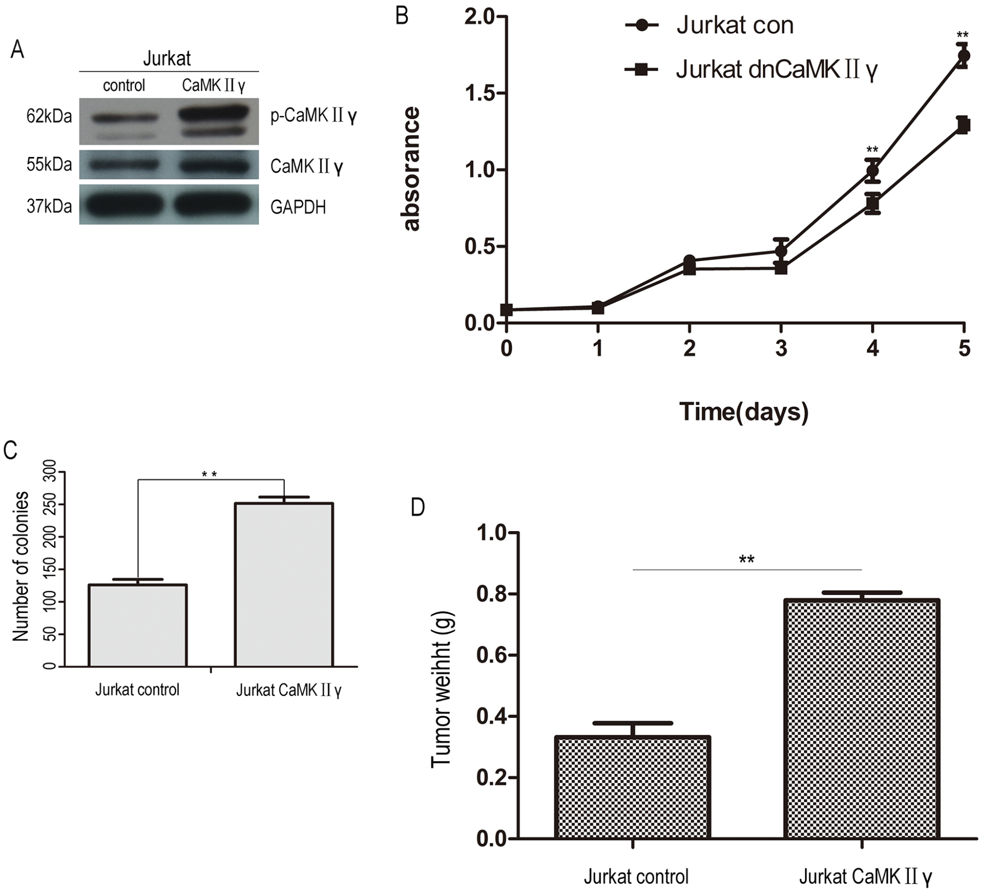


**Supplementary Figure 2: (A)** Jurkat CaMKIIγ and Jurkat control cells lysates were subjected to Western blots with CaMKIIγ, p-CaMKIIγ, GAPDH was used as a loading control. **(B)** Jurkat dnCaMKIIγ (T287A) and Jurkat control cells were seeded in 96-well plates. The CCK-8 assays were performed at the indicated times (**p<0.01). **(C)** Comparisons of colony numbers between control and CaMKIIγ overexpression in Jurkat cells (**p<0.01). **(D)** Comparisons of tumor weights between Jurkat control and Jurkat CaMKIIγ in NSG mice (**p<0.01).

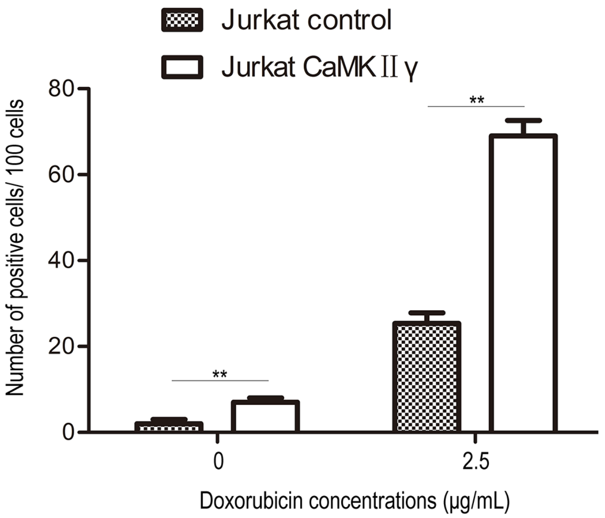


**Supplementary Figure 3: Comparisons of numbers of γ-H2AX-positive cells between Jurkat control and Jurkat CaMKIIγ cells (**p<0.01).**

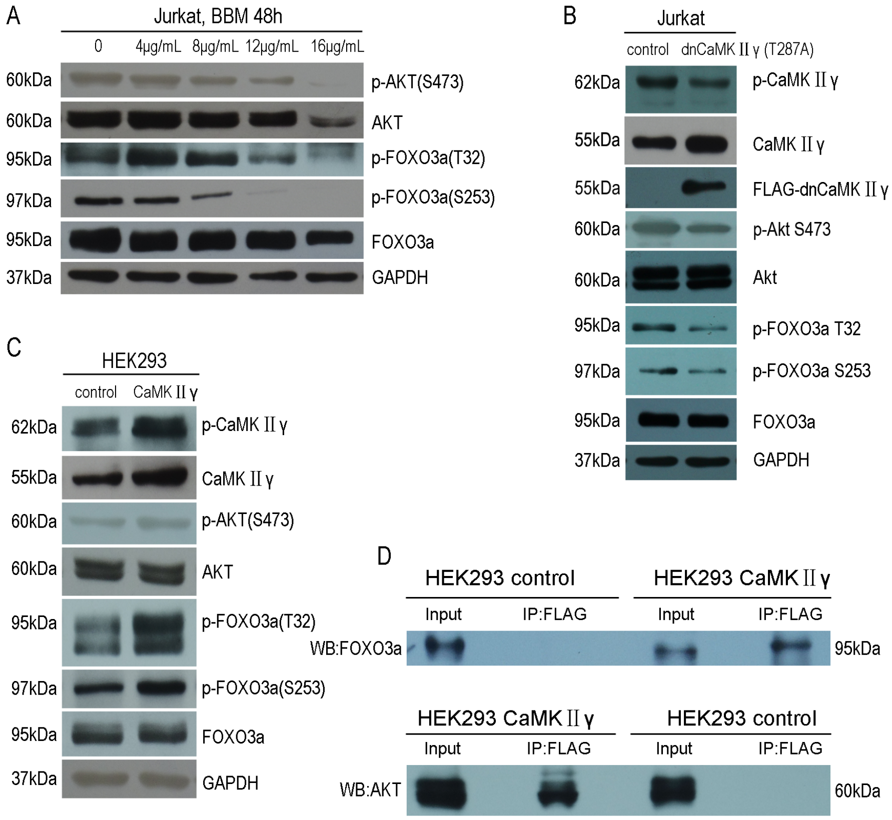


**Supplementary Figure 4: (A)** Jurkat cells lysates treated with BBM for 48 hours were subjected to Western blots used the AKT, p-AKT(S473), FOXO3a, p-FOXO3a(T32), p-FOXO3a(S253) antibodies. **(B)** Jurkat dnCaMKIIγ (T287A) and Jurkat control cells lysates were subjected to Western blots with CaMKIIγ, p-CaMKIIγ, AKT, p-AKT (S473), FOXO3a, p-FOXO3a (T32), p-FOXO3a (S253) antibodies. **(C)** HEK293 CaMKIIγ and HEK293 control cells lysates were subjected to Western blots with CaMKIIγ, p-CaMKIIγ, AKT, p-AKT (S473), FOXO3a, p-FOXO3a (T32), p-FOXO3a (S253) antibodies. In (A-C) GAPDH was used as a loading control. **(D)** HEK293 CaMKIIγ and HEK293 control cells lysates were immunoprecipitated with FLAG antibody. The immunoprecipitates were then subjected to Western blots with AKT and FOXO3a antibodies.
